# Nutritional Wasting Disorders in Sheep

**DOI:** 10.3390/ani11020501

**Published:** 2021-02-15

**Authors:** Javier Asín, Gustavo A. Ramírez, Mauricio A. Navarro, Akinyi C. Nyaoke, Eileen E. Henderson, Fábio S. Mendonça, Jéssica Molín, Francisco A. Uzal

**Affiliations:** 1 California Animal Health and Food Safety Laboratory (CAHFS), San Bernardino Branch, University of California, Davis, CA 95616, USA; mnavarrob@ucdavis.edu (M.A.N.); canyaoke@ucdavis.edu (A.C.N.); eehenderson@ucdavis.edu (E.E.H.); fauzal@ucdavis.edu (F.A.U.); 2 Animal Science Department, University of Lleida, 25198 Lleida, Spain; gustavo.ramirez@udl.cat (G.A.R.); jessica.molin@udl.cat (J.M.); 3 Laboratory of Animal Diagnosis, DMFA/UFRPE, Recife, Pernambuco 52171-900, Brazil; fabio.mendonca@ufrpe.br

**Keywords:** sheep, nutritional management, acidosis, mineral deficiencies, wasting

## Abstract

**Simple Summary:**

Nutritional management is one of the most important factors to ensure adequate productivity and to prevent wasting in sheep flocks. Food needs to be offered in enough quantity and quality to avoid deficiency issues. Motility and metabolic disorders, such as subacute ruminal acidosis, may also lead to emaciation. A combination of a detailed flock history, clinico-pathologic findings, and ancillary tests is necessary to diagnose these conditions. Treatment approaches range from reinstating the levels of the depleted dietary compound to modifications in management practices.

**Abstract:**

The different ovine production and breeding systems share the cornerstone of keeping a good body condition to ensure adequate productivity. Several infectious and parasitic disorders have detrimental effects on weight gains and may lead to emaciation. Flock health management procedures are aimed to prevent such conditions. Nutritional management is equally important to guarantee adequate body condition. Persistent bouts of low ruminal pH due to excess concentrate in the diet may lead to subacute ruminal acidosis. Pre-stomach motility disorders may also lead to ill-thrift and emaciation. An adequate mineral supplementation is key to prevent the effects of copper, selenium, and other micronutrients deprivation, which may include, among others, loss of condition. This review elaborates on the clinico-pathologic, diagnostic, and therapeutic aspects of some of these conditions, and highlights the necessity of considering them as contributors to states of wasting in sheep flocks.

## 1. Introduction

Sheep are reared in a broad variety of geographical locations and production systems across the world. Sheep flocks may have different purposes, including meat, milk, and wool production, or, most frequently, a combination of two or three of them [1]. Irrespective of the breeding purpose of a flock, maintaining a good body condition is a basic pillar of the nutritional and health management in order to maximize productivity [2].

Many diseases may compromise sheep body condition, affecting either directly or indirectly food intake and/or nutrient assimilation. Perhaps the most well-known amongst these processes are those related to infectious and parasitic agents [3,4,5,6,7], which over the years have generated many research and diagnostic efforts to prevent their harmful effects. There are also several nutritional, metabolic, and digestive motility disorders that may disrupt nutrient assimilation and cause loss of condition. A correctly managed diet, which ensures that enough quality food is offered to the animals, is key to prevent wasting due to starving and/or imbalanced mineral intake.

Here we review some of these disorders with especial emphasis on their clinical signs, lesions, diagnostic approaches, and treatments.

## 2. Metabolic, Motility, and Management Disorders

### 2.1. Malnutrition

An adequate diet is the first requirement that needs to be fulfilled in order to prevent states of wasting in sheep. Feed needs may vary depending upon the purpose of the flock but, in general, supplementation is needed in periods of scarcity, such as winter months [8]. This is especially relevant in purely extensive systems. Malnutrition has a prominent impact on the reproductive function [9], and thus the pre-mating body condition is one of the most important parameters to be controlled in the majority of systems [10].

Emaciation in animals may be the result of two different mechanisms: cachexia, which is cytokine-mediated and associated with endogenous disease; and starvation, which is due to a reduction in the caloric intake and generally related to exogenous circumstances (e.g., food deprivation and/or scarcity, adverse environmental or management conditions, etc.) [11,12]. Unfortunately, there is no definitive parameter available to differentiate emaciation due to exogenous circumstances (starvation) from cachexia, and a good history focused on the dietary and health management of the flock should be the first diagnostic approach to take. Then, if deemed necessary, multiple diagnostic tests that rule out endogenous disease may be applied [12].

Body condition assessment is valuable to determine the nutritional status of sheep. Animals are palpated on the loin, immediately behind the last rib, and the prominence of the spinous and transverse vertebral processes is evaluated subjectively by estimating the amount of muscle (*longissimus dorsi*) and fat covering these prominences. A six-tier system is commonly used, 0 being totally emaciated and 5 being obese [13]. An adequate body condition score should range between 2 and 4, depending upon the purpose of the flock, breeding stage, and geographical location [10].

The postmortem examination of an emaciated sheep should be focused on ruling out lesions associated with endogenous causes of disease, e.g., Johne’s disease, maedi-visna, endoparasitosis, poor dentition, etc. [3,4,6,14,15], that may have prompted such status. Grossly, a sheep that died from chronic starvation usually presents with depletion and serous atrophy of fat deposits. Serous atrophy (also known as gelatinous transformation or myxomatous degeneration) occurs as a result of mobilization of fat reserves, which leads to a watery to jelly-like consistency of the remaining adipose tissue deposits [14,16,17]. Affected regions usually include epicardial, pericardial, perirenal, mesenteric, omental, and bone marrow fat deposits [14,18]. Effusions in body cavities, including pericardial sac, abdomen, and thorax, as well as a degree of subcutaneous edema, may also be detected and are likely due to hypoproteinemia. In extreme cases, muscle atrophy ensues, and the liver may appear small, likely due to loss of trophic stimuli [18]. Microscopic examination may help to demonstrate endogenous causes of emaciation that were missed grossly, but generally does not add much information in cases of starvation. Atrophic adipocytes over a faintly basophilic to amphophilic, extracellular matrix are detected in the depleted fat stores. This matrix stains light blue with Alcian blue, and is thus believed to be made of mucopolysaccharides rich in hyaluronic acid [19].

Measuring the percentage of fat in the bone marrow of a long bone by quantitative methods can provide with an unbiased evidence of adipose tissue depletion [20,21]. The average percentage of fat in the femoral bone marrow for animals in good body condition is >80%, whereas emaciated individuals present with values of less than 20% [20]. Vitamin and mineral imbalances may occur concomitantly in chronically emaciated sheep, thus evaluating their levels adds information to the clinico-pathologic picture [22]. In addition, undernourished sheep are predisposed to parasitosis by gastrointestinal nematodes [17,23], which may contribute further to loss of condition. Sheep tend to respond well to the reestablishment of an adequate diet, thus correction of the deficient management procedures is the main therapeutic approach in flocks with malnutrition [22].

### 2.2. Subacute and Chronic Ruminal Acidosis

Rumen acidosis (ruminal acidosis, rumen lactic acidosis, grain overload, rumenitis) is a metabolic disorder that occurs after the ingestion of readily fermentable carbohydrates and their subsequent rapid ruminal fermentation [24]. The disease occurs frequently in cattle and small ruminants [18,24,25], and may be acute, sub-acute, or chronic [25].

Ruminal acidosis associated with ingestion of excess carbohydrate in sheep is usually associated with intensive breeding systems [18,25]. While acute acidosis usually causes death, sub-acute and chronic acidosis are responsible for loss of production. Sub-acute and chronic acidosis cause rumenitis, which is also important as a port of entry for *Fusobacterium necrophorum* and fungi, thus leading to secondary ruminal and hepatic infections. Primary tympany (frothy bloat) is another complication of rumenitis [18].

**Figure 1:**
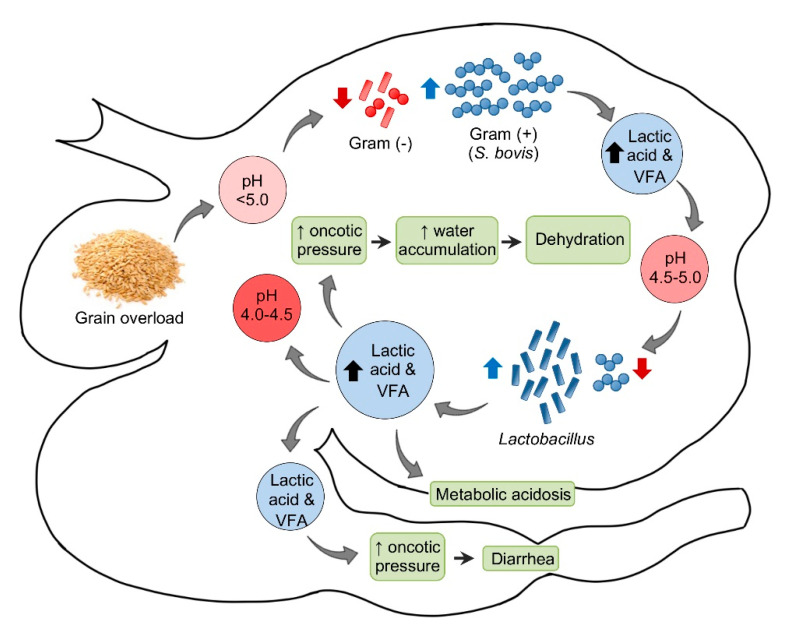
Pathogenesis of ruminal acidosis. VFA = Volatile fatty acids.

The pathogenesis of acute ruminal acidosis is usually the consequence of sudden ingestion of excess carbohydrate in animals not accustomed to it (Figure 1). These carbohydrates include, amongst others, grain, bread, waste baked goods, brewers’ waste, root crops, and apples. The amount of carbohydrate necessary to produce acidosis is highly variable and the individual tolerance increases when those carbohydrates are introduced progressively. Hence, the sudden introduction or increase of carbohydrates are more critical than the actual amount [18,25].

In sheep, the normal ruminal pH is 5.5–7.5, depending on the diet, with Gram-negative bacteria being the predominant component of the ruminal microbiota. Very soon after consumption of a large amount of carbohydrate to an unaccustomed animal, the ruminal pH begins to drop. This initial decrease in pH is mainly the consequence of an increase in dissociated volatile fatty acids (VFA). Most Gram-negative bacteria and protozoa, which are very sensitive to changes in pH, die when the pH reaches 5.0 or below. When this happens, *Streptococcus bovis* and other streptococci start to proliferate rapidly and produce lactic acid, which reduces the pH to 5.0–4.5. At this point, there is a switch in the ruminal microbiota, characterized by an increase in lactobacilli and a decrease in streptococci. In fatal cases, the pH of the rumen content may fall as low as 4.0–4.5 [18].

Saliva secretion also stops, which eliminates the important buffering effect of this substance. As the concentrations of the non-dissociated VFA, lactic, propionic, and butyric increase, these acids act on receptors that mediate inhibition of reticuloruminal motility via a vagovagal reflex, leading to ruminal atony. The ruminal oncotic pressure increases because of the increased concentration of these ruminal organic acids, mainly lactate. The consequence of this is accumulation of water in the rumen and severe dehydration with reduction of plasma volume, hemoconcentration, anuria, and circulatory collapse. When lactate passes through the intestine, the oncotic pressure of the intestinal content also increases producing liquid content in this part of the intestinal tract and thus leading to diarrhea. Lactate is also absorbed from the rumen, and possibly from the intestine, leading to metabolic acidosis. As a consequence of general dehydration, there is an increase of serum protein, urea, inorganic phosphorus, lactate, pyruvate, and liver enzymes. Most of the normal ruminal flora and fauna die as a result of the low pH. Animals that survive acute ruminal acidosis need to have their ruminal microbiota re-established. This may occur by therapeutic ruminal content transplant or by contact with feces of unaffected animals [18]. Sub-acute and chronic acidosis have a similar pathogenesis, although the drop in pH tends to be milder and persistent, leading to physical damage of the ruminal mucosa and submucosa.

Clinical signs of acute ruminal acidosis may be observed within 12 h after ingestion of concentrate, and they include lethargy, anorexia, bruxism, nasal discharge, head pressing, ataxia, hyperpnea, recumbency, dehydration, scleral injection, muscle twitching, tachycardia, fluid abdominal sounds, reduced or absent motility of the rumen, mild colic, diarrhea and pH reduction to 5 or 6. In severe cases, shock and coma leading to death may also occur [24,26].

In subacute and chronic ruminal acidosis, clinical signs include reduced or cyclic feed intake, decreased milk production, reduced fat, poor body condition and diarrhea. Additionally, unusually high rates of culling or unexplained deaths may be noted in the flock. Diarrhea is inconsistently seen [27]. Laminitis is a traditionally suggested sequel of all forms of rumen acidosis [24,25]; however, the pathogenesis for acidosis and laminitis has been recently disputed in cattle, since there seems to be lack of clear evidence to support the classic laminitis hypothesis [25,28]. Further research into the mechanistic basis of the association between ruminal acidosis and laminitis in sheep is necessary.

The gross findings of acute ruminal acidosis are not specific and are characterized by signs of dehydration and hypoxia, including sunken eyes, dense and dark blood, and general venous congestion. Soon after the ingestion of large amounts of carbohydrates, the rumen content has a porridge-like appearance with a distinct fermentative odor. There may or may not be a large amount of grain or other sources of starch, but care should be taken not to overlook finely ground concentrate. In sub-acute or chronic cases of acidosis, the ruminal content may appear more or less normal, but the intestinal contents remain watery. There may be a poorly defined slight blue discoloration in the ventral sac of the rumen and reticulum and in the omasum, visible through the serosa. When the epithelium is detached, the lamina propria is patchy hyperemic. In some cases, the epithelium appears to have undergone fixation due to low pH, and is difficult to peel [18]. Patches of flat, white ruminal mucosa devoid of papillae, and multiple abscesses or foci of necrosis may be evident in the liver. Interpretation of the post-mortem rumen pH may be challenging. Some authors consider that because ruminal fermentation continues after death, the pH of rumen contents declines postmortem [29].

**Figure 2:**
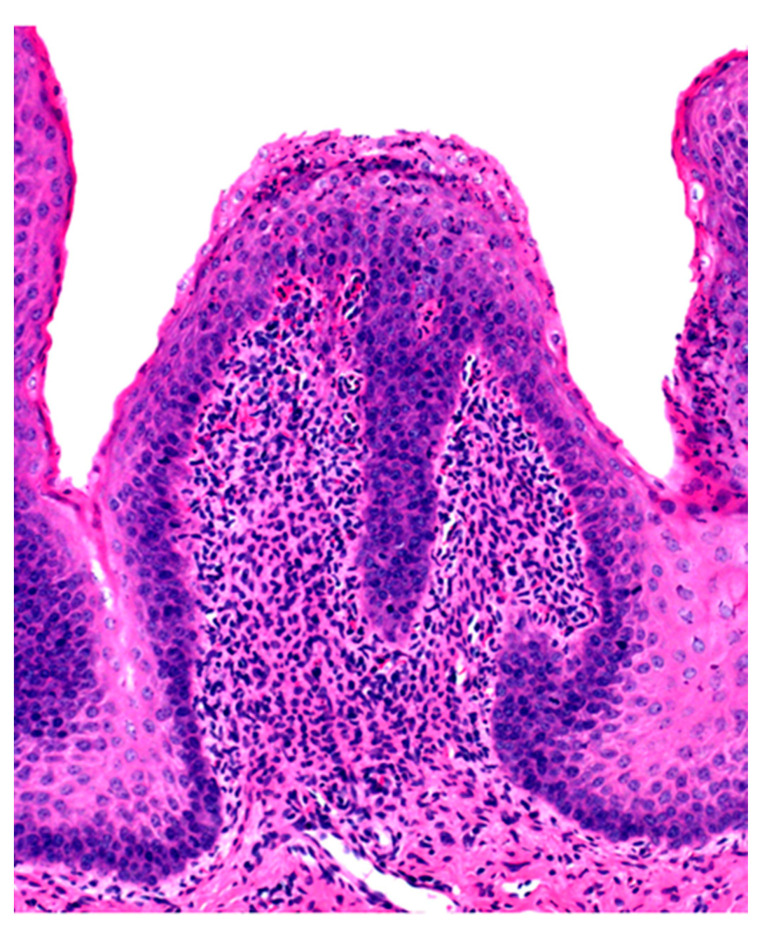
Ruminal papilla with intraepithelial ne trophilic aggregates and pleocellular inflammation in the lamina propria.

Microscopic changes are usually absent in the rumen of animals dying of acute rumen acidosis, unless pre-existing subacute lesions were present before the onset of acute disease. Microscopic examination of the rumen in cases of sub-acute or chronic acidosis reveals lesions consistent with chemical rumenitis, including enlarged ruminal papillae, cytoplasmic vacuolation of the epithelial cells often leading to vesiculation, and a mild to marked neutrophilic infiltration in the mucosa and submucosa (Figure 2). There may be multifocal erosion and ulceration of the superficial mucosa. Absence of protozoa is consistent with chemical rumenitis, but is also influenced by the interval between death and the postmortem examination. Similar changes may be detected in the other pre-stomachs. Apart from general congestion, there are no specific changes in other organs [18].

For the diagnosis of acidosis in the live animal, measurement of the rumen fluid pH is helpful. Collection of rumen fluid may be performed by rumenocentesis or by aspiration through a tube. A pH of 5.5 or less is strongly suggestive of rumen acidosis [30]. However, animals accustomed to a high grain ration may have rumen pH close to 5 [31]. On microscopic examination of rumen fluid, the numbers of protozoa are significantly reduced or absent, and Gram-positive bacteria predominate [26].

Blood gas analysis of affected small ruminants may help to establish a diagnosis of acidosis and it is characterized by changes compatible with metabolic acidosis. There may also be elevation of hepatic enzymes in blood. The postmortem diagnosis of acute ruminal acidosis may be difficult because there are usually nonspecific gross or microscopic lesions. The ruminal pH is diagnostic when it is low (<5.0), but a normal pH does not preclude a diagnosis of ruminal acidosis, because the ruminal pH tends to vary after death. If acute acidosis occurred in animals that were previously suffering sub-acute or chronic acidosis, there may be lesions of rumenitis, but this is only an indication of pre-existing lesions and not of acute acidosis [18]. In sub-acute and chronic acidosis, neutrophilic or pleocellular rumenitis is diagnostic for those conditions [18].

### 2.3. Vagal Indigestion Syndrome

Vagal indigestion syndrome (also referred to as vagus indigestion or Hoflund syndrome) is characterized by disturbances in the passage of ingesta from the forestomachs to the abomasum [32]. This syndrome is seen most commonly in dairy cows, less commonly in bulls and beef cattle, and is relatively uncommon in sheep and goats. Affected animals often present with a history of inappetance and weight loss, abdominal distension, scant feces, and hyper- or hypomotility of the rumen/reticulum.

The term vagal indigestion comes from a study conducted by Hoflund in cattle in the 1940s [33]. In this study, Hoflund performed an experimental vagotomy, which resulted in reduced outflow of ingesta from the forestomachs to the abomasum. A study by Malbert and Ruckebusch (1989) [34] demonstrated that reticuloruminal contractions in sheep ceased immediately following bilateral vagotomy; whereas abomasal contractions occurred more frequently. Two weeks following vagotomy, the strength but not the duration or frequency of reticuloruminal contractions were similar to those noted prior to the procedure. The role of vagal nerve injury in many cases clinically diagnosed as “vagal indigestion” is not known.

Classically, vagal indigestion syndrome is categorized by the region of the gastrointestinal tract affected [35]. Type 1 vagal indigestion is characterized by a failure of eructation resulting in gas distension of the rumen and reticulum. Type 2 vagal indigestion is associated with failure of rumen outflow; this is the most common type of vagal indigestion and is often associated with traumatic reticuloperitonitis in cattle and less commonly in small ruminants. Any cause of focal peritonitis, intraabdominal abscess, and/or adhesions can result in vagal indigestion. Type 3 vagal indigestion results from abomasal outflow failure with reflux of abomasal contents into the forestomachs. Type 4 vagal indigestion is less well defined and is thought to be associated with disruptions in pyloric outflow or generalized ileus; this type of vagal indigestion typically occurs during late pregnancy. Various neoplasms are also thought to play a role in several types of vagal indigestion either through mechanical obstruction (e.g., fibropapilloma) or functional disturbances (e.g., lymphoma). A diagnosis of vagal indigestion at necropsy typically involves evidence of abnormal motility of the forestomach/s and/or abomasum and either peritonitis, intraabdominal abscesses, or neoplasia with involvement of the vagus nerve [18].

There are relatively few documented cases of vagal indigestion syndrome in sheep in the literature. In one case report, a mineralized *Cysticercus tenuicollis* cyst was found in the wall of the reticulum of a six-year-old ewe [36]. The cyst was associated with intraperitoneal adhesions and appeared to involve the ventral branch of the vagus nerve. This animal presented with intermittent abdominal distension and weight loss. Intraperitoneal abscesses associated with caseous lymphadenitis have also been documented in association with vagal indigestion in goats [37].

### 2.4. Abomasal Emptying Defect

A syndrome characterized by marked distention and impaction of the abomasum of sheep has been referred to as abomasal emptying defect (AED), also known as abomasal impaction, and abomasal dilatation and emptying defect [38]. Most cases have been reported in Suffolk sheep, which may be particularly predisposed to this condition, although Hampshire, Dorset, and Texel sheep can also be affected [38,39,40,41]. Though the cause is not known, a presumable toxic-related, acquired dysautonomia in association with a genetic predisposition, has been suggested [18,38]. An unidentified viral component has also been proposed but not proven to date [38].

Commonly, AED occurs sporadically; however, a few outbreaks have been reported in which no inheritance patterns were determined [38,42]. Affected animals are commonly adults (2–6 years), with no evident sex predisposition. The disease is clinically characterized by right ventral abdominal distention, anorexia, and progressive weight loss [38,43]. The clinical course can last days or up to several months. Ruminal chloride concentrations are elevated, which may indicate abomasal reflux [43]. Although treatment with laxatives, motility modifiers, and abomasotomy have been attempted in some cases, the prognosis of AED is almost invariably poor [42,43].

At necropsy, the abomasum is markedly distended with large amounts of dry ingesta. In most severe cases, dilation of the omasum and esophagus may occur. Microscopic lesions may be found in the anterior celiaco-mesenteric ganglion, and they are characterized by the presence of scattered chromatolytic and necrotic neurons with minimal or no leukocytic infiltration, suggesting, as previously mentioned, a form of acquired dysautonomia [18,38]. Reduction in the number of myenteric or submucosal ganglia throughout the abomasum or omaso/abomasal junction has been reported in one animal [38].

## 3. Mineral Deficiencies

This section enquires into different mineral/trace element deficiencies that may contribute to states of ill-thrift and wasting. Most of these disorders often present with a variety of clinical signs and lesions that may facilitate the diagnosis. Indices to evaluate the risk of deficiency disorder have been established for some of these minerals [44,45], although these values may vary depending on the geographical location, diet, method of measurement, etc.

### 3.1. Copper Deficiency

Copper (Cu) is an essential component of many enzymes, and it is vital for a proper development and maintenance of several organ systems, such nervous, integumentary, skeletal, hematopoietic, immune, and digestive systems [44,46].

Cu deficiency in sheep may be primary, due to decreased intake, or secondary, due to altered absorption, reduced tissue availability, or enhanced excretion [44,45,46,47]. In ruminants, Cu has a complex array of interactions with dietary molybdenum (Mb) and sulfur that, when present in excess, will lead to production of molybdates and thiomolybdates, which bind Cu and decrease its absorption and utilization [44,46,48]. Other elements, such as zinc (Zn), iron, cadmium, and lead may act as Cu-antagonists and interfere with its bioavailability. Gastrointestinal parasites are another cause of conditioned hypocuprosis [44,46,49].

The pathogenesis of Cu deficiency is related to decreased activity of many Cu-dependent enzymes in several tissues. Cu is a component of several enzyme systems that are essential for neural function [44,47] and, therefore, lesions are characterized by primary neuroaxonal degeneration with secondary myelin loss [44,47,50]. Impaired function of mitochondrial cytochrome C oxidase, superoxide dismutase (SOD), and ceruloplasmin have been proposed as the most important molecular bases of the disease, since there is suppression of mitochondrial respiration and phospholipid synthesis, with possible neuronal damage by superoxide radicals [47,50]. Loss of embryonic cells during brain development has also been involved in the pathogenesis of congenital cerebral cavitation [50].

In addition to nervous tissue, many other systems may be affected by Cu deficiency. Poor wool quality and hypopigmentation are caused by imperfect oxidation of sulfhydryl groups in prekeratin, and by decreased melanogenesis due to depressed tyrosinase activity, respectively [51]. Impaired production of ceruloplasmin and hephaestin reduce gastrointestinal iron absorption, which leads to decreased hemoglobin synthesis, altered maturation of erythroid precursors, and eventually anemia after prolonged periods [44,45]. Moreover, increased oxidative stress due to Cu depletion in erythrocytes and muscle fibers can lead to hemolysis secondary to Heinz body formation, as well as to muscle necrosis [44,52] (see Section 3.3. Selenium/Vitamin E Deficiency). Decreased functions of Cu-dependent enzymes on intestinal mucosa interfere with digestion, motility, and inflammatory responses to parasites, which might account for growth retardation and increased susceptibility to gastrointestinal parasites [44].

Fragility of connective tissue and abnormal bone matrix formation are associated with decreased collagen cross-linking due to reduced lysyl oxidase activity and with impaired function of cytochrome C oxidase in osteoblasts [44,53,54]. Altered SOD and catalase activity in granulocytes impair innate immunity. Infertility, although probably multifactorial in cases of secondary Cu deficiency, may be due in part to increased blood oxythiomolybdates, which interfere with the release of luteinizing hormone [44].

Clinical signs of Cu deficiency in sheep depend on the stage of development at the time of deprivation. The disease manifests as a congenital form or swayback, which is evident at birth, or as a delayed form, known as enzootic ataxia. In lambs, the congenital form is more common and clinical manifestations are more severe than in the delayed form [45,47,50]. Lambs with swayback are born weak and with severe ataxia, occasional blindness and/or deafness, progressive recumbency, and they usually die within the first days of life; in some cases, stillbirths occur [45,47,55]. Delayed forms are characterized by development of clinical signs at any time between 1 week and up to six months of age. Affected lambs show progressive flaccid paraparesis and ataxia, with a tendency to sway on their hind legs, followed by recumbency and death within six months [45,47,55]. Changes in wool quality and pigmentation are early manifestations of the disease in growing lambs and adult sheep. There is loss of wool crimp, and diminished tensile strength and elasticity, known as “string or steely wool”. This is often accompanied by wool hypopigmentation in black sheep, which may appear as intermittent bands of light-colored wool reflecting Cu-deficiency periods [45,51]. Other less specific clinical manifestations include increased tendency to bone fractures, poor appetite and growth retardation, hypochromic microcytic (lambs) or macrocytic (adults) anemia, increased susceptibility to microbial infections and, in Cu-depleted ewes, infertility often associated with small dead fetuses [44,45].

Unequivocal gross lesions of Cu-deficiency in sheep are mostly detected in lambs with congenital swayback. Approximately 50% of the cases present with bilateral and symmetrical softening or cavitation of the periventricular cerebral white matter (corona radiata and centrum semiovale) with associated ventricular distension [47,50,56]. Lesions might be restricted to the occipital lobe or affect the entire hemisphere. In growing animals and dams, a combination of the above-mentioned fleece alterations, long bone fractures, and emaciated carcasses with reduced fat stores can be observed [44,45].

Microscopic lesions are present in lambs with congenital and delayed forms. Cavitating cerebral lesions in the congenital form consist of severe edema with mild fibrillary astrogliosis and myelin paucity [47,50]. There may be myelin degradation and sparse gitter cells. Laminar neuronal necrosis, sometimes with calcified neurons, may be detected in the cerebral cortex overlying areas of white matter rarefaction or cavitation [56]. Similar microscopic changes in the gray and white matter of the spinal cord and brainstem may be detected in both congenital and delayed forms. There is extensive pallor due to Wallerian-like degeneration in the dorsolateral aspect of the lateral funiculi (spinocerebellar tracts) and the ventromedial aspect of ventral funiculi. [47,50,56]. In the gray matter, there is conspicuous neuronal degeneration, characterized by central chromatolysis or necrosis, which affects spinal cord motor neurons (particularly at the intumescences) and specific brainstem nuclei [47,50,56]. Ultrastructurally, chromatolytic neurons show ribosomal depletion and aggregates of enlarged mitochondria, together with accumulation of phosphorylated neurofilaments [47,56]. Wallerian-like degeneration might be observed in ventral spinal nerve rootlets and spinal nerves [47,50,56]. In contrast to kids, a very small number of lambs may have cerebellar lesions characterized by necrosis of Purkinje cells, depletion of internal granule cells, and degeneration in folial white matter. A variant form reported in lambs with delayed presentations encompasses acute cerebral edema with occasional small gelatinous or cystic foci at the corticomedullary junction [45,47,56]. Microscopic lesions of osteoporosis in long bones, and to a lesser extent in costo-chondral junctions, are most prominent in the metaphyseal region and include severe reduction in the number of osteoblasts with narrowing of the cortices, decreased bone trabeculae in the central part of the metaphysis, and absence of bone matrix deposition with rare evidence of complete cessation of bone growth [53,54].

A tentative diagnosis of swayback is easily achieved by the detection of compatible gross lesions and histological examination of the CNS. Confirmation requires detection of low Cu concentrations in these tissues [47]. Otherwise, with exception of swayback, the non-specific clinical signs of Cu deprivation make hypocuprosis hard to diagnose. In these cases, diagnosis should rest upon three criteria: (i) Presence of clinical or subclinical signs; (ii) biochemical evidence of Cu levels below reference values in animal tissues (i.e., blood, liver); and (iii) improvement of productive parameters after Cu supplementation [44]. Hepatic Cu concentration is the best indicator, as it provides a direct measure of the main storage compartment in the organism. Blood Cu levels should be measured in plasma, but they only fall after there has been significant depletion of liver reserves [44,45]. Low SOD activity in erythrocytes indicates a prolonged deficiency and might be associated with impaired function and growth. Pasture analysis may be used as an additional diagnostic tool to tissue analysis; it should cover Cu, Mb, and sulfate, and the relative concentrations of each are as important as the individual values [44,45].

Lesions caused by Cu deficiency are irreversible. Therefore, all efforts should be centered on preventive measures. A variety of prophylactic methods to provide livestock with enough Cu exists, and their differences in efficacy and suitability have been largely reviewed [44,45]. Preventive measures include the use of pasture fertilizers, oral dosage, dietary supplementation, and subcutaneous or intramuscular injections of chelated Cu, but other strategies exist [44]. Oral dosing in pregnant ewes, one and two months before lambing, have been widely used to prevent swayback, but does not necessarily protect lambs from growth retardation [44,45]. Oral administration of Cu to newborn lambs may be effective to prevent delayed onset forms, and is recommended in all suckling animals after the first clinical case occurs in a flock. Subcutaneous injections can also be used but are less preferred, as they may be associated with local reactions. Oral administration of Cu oxide wire particles in pellets, which lodge in the abomasum, is an effective method to guarantee slow-release Cu supplementation to suckling and weaned lambs and/or adult sheep; however, over-correction should be carefully avoided to prevent toxicity [44,45].

### 3.2. Cobalt Deficiency

A regular dietary cobalt (Co) supply is essential for survival, health, and efficient production. Sheep are particularly susceptible to Co deficiency due to their high S-amino acid requirements for wool growth. Co deficiency is of economic importance in weaning lambs, as it causes an ill-thrift syndrome, clinically indistinguishable from that caused by starvation, occasionally associated with hepatic disease, and known as ovine white liver disease (OWLD) [44,45].

The major cause of Co deficiency in sheep is inadequate dietary supply. Clinico-pathologic consequences derive from insufficient vitamin B12 synthesis by ruminal microorganisms, leading to decreased tissue availability of its coenzymes, methylcobalamin and deoxyadenosylcobalamin, which assist methionine synthase and methylmalonyl coenzyme A mutase, respectively [44].

Methionine synthase is a methyl transferase involved in the synthesis of methionine from homocysteine, an essential amino acid with important roles in DNA and protein synthesis. Reduced methionine availability causes ineffective production of cells, particularly those with rapid turnover and, consequently, impaired normal growth and development [44,45]. Moreover, homocysteine accumulation increases lipid peroxidation in the liver, which is considered an important mechanism involved in the pathogenesis of OWLD [44,57]. Methylmalonyl coenzyme A mutase performs a key role in the intermediary energy metabolism of ruminants, facilitating the metabolism of propionate via succinate, which serves for gluconeogenesis [44,45]. Metabolic abnormalities resulting from alterations of this enzymatic pathway and accumulation of intermediary metabolites in plasma, such as methylmalonic acid and propionate, are important in the pathogenesis of OWLD and other alterations related to Co deficiency. Methylmalonic acid becomes incorporated into branched-chain fatty acids, which may alter lipid composition in the liver, and also inhibits the oxidation of fatty acids mobilized to supply the energy deficit caused by anorexia in affected animals [44,45]. Poor reproductive performance in ewes has been attributed to impaired gluconeogenesis in addition to generalized poor body condition [44,58]. Increased propionate levels in the portal blood flow induce powerful satiety signals in sheep and has been suggested as a cause of anorexia in Co-depleted lambs [44,45], but this hypothesis in considered unlikely by others [59].

Clinical signs of Co deficiency are most commonly observed in weaned lambs at pasture during late summer/autumn. Affected animals show lethargy, reduced appetite, growth retardation, poor wool quality, small size, and poor body condition despite adequate nutrition. Lachrymation and wool alterations might be the earliest visible abnormalities [44,45,60]. In chronic stages, animals develop anemia with pallor of the mucous membranes, and progressive anorexia with marked weight loss, emaciation, muscular wasting, pica and decreased wool growth; without intervention, death ensues [44,45]. Additionally, clinical signs related to hepatic dysfunction such as icterus, photosensitization, or neurological signs secondary to hepatic encephalopathy might be present in animals affected by OWLD, particularly in the acute stages [45,61,62]. An increased susceptibility to gastrointestinal parasitic infections has been also reported in Co deficient lambs [44]. In adult ewes, Co deficiency during pregnancy may cause infertility, poor mothering, and a reduction of the viability of the offspring [44,58].

In severely affected animals, the carcass is extremely emaciated, without visible fat deposits and generalized muscle atrophy [60,62,63]. In the acute stage of OWLD, liver is grossly enlarged, pale and friable [62,63,64]. The most important histological lesions associated with Co deficiency in lambs are detected in the liver. Hepatic changes include centrilobular to diffuse lipidosis, widespread hepatocyte disassociation and tissue architecture disruption, hepatocellular apoptosis, intracytoplasmic ceroid, and lipofuscin accumulation in hepatocytes and Kupffer cells, and periportal ductular reaction with variable degrees of mononuclear inflammation and, in chronic stages, periportal fibrosis [60,62,63,64]. Ultrastructural changes include mitochondrial degeneration and proliferation of the smooth endoplasmic reticulum [60,62]. Splenic hemosiderosis is common but usually mild [44,61]. Reduced cellularity of sternal bone marrow and erythroid hypoplasia is present in chronically affected animals with anemia [44,45]. Occasionally, polioencephalomalacia and/or spongy degeneration of cerebral white matter might be present due to hepatic encephalopathy [53,62,64].

The diagnosis of OWLD relies on a combination of clinical signs, pathological changes in the liver, and evidence of decreased hepatic levels of vitamin B12 [44,45]. Mild Co deprivation is impossible to diagnose clinically because the unthrifty appearance is indistinguishable from other causes of emaciation [44]. Most common biochemical indices to assess Co and vitamin B12 status in the flock include serum and hepatic levels of vitamin B12, and serum levels of methylmalonic acid and homocysteine, but results should be interpreted with caution as are influenced by different factors [44,45]. At the onset of the lactation, ewes may show increased methylmalonic acid without changes in vitamin B12 levels in the serum or liver, possibly induced by higher propionate turnover [44,59]. Plasma methylmalonic acid is less useful in suckling lambs, in which indicators of methylcobalamin dysfunction such as homocysteine are preferred [44,45]. Clinico-pathologic parameters, such as plasma levels of hepatic transaminases and other markers of hepatic dysfunction, might be useful to determine hepatic injury and to establish the course of the disease [44,45,63,64]. Anemia is indicative of chronic Co depletion [44,45,63]. Nevertheless, improvement of appetite and growth rate in response to Co therapy is the most reliable diagnostic criterion [44].

Several continuous and discontinuous methods are available for treatment and prevention of Co deficiency, and their application depends on economic, dietary, and husbandry factors [44,45]. Continuous methods based on dietary supplementation are preferred. Amongst discontinuous methods, oral administration of Co-containing, slow-release pellets of soluble gas boluses provide a steady supply for many months [44,45]. Oral drench of Co sulphate or vitamin B12 injections confer the most immediate response, but the duration of these effects is short and, therefore, the required high frequency of such treatments makes them unsuitable as prophylactic methods [44,45,63].

### 3.3. Selenium/Vitamin E Deficiency

Selenium (Se) is an essential element that interferes through selenoproteins (SePs) in many physiological processes of livestock [44,65]. In many instances, Se deficiency must be addressed with vitamin E deficiency. Se and/or vitamin E deficiency in sheep are well-known causes of nutritional myopathy (NM) or white muscle disease, but subclinical forms with ill-thrift and reproductive issues may be the most important manifestations of Se deficiency in economic terms [44,45].

The major cause of Se and/or vitamin E deficiency is inadequate dietary supply [44,65]. Se deficiency occurs in many regions, particularly in those with soils of granitic or volcanic origin, and in areas of heavy rainfall. It can also be associated with intensive farming practices [44,45]. Vitamin E deficiency is independent of soil type. Green pastures and fresh legumes are good sources; therefore, vitamin E deficiency is widespread in weaner sheep flocks over long, dry summer and autumn, when animals are fed on dry feed, hay, and grain with little or no access to green pastures for long periods. Prolonged storage of feedstuffs also causes important loss of vitamin E content [44,45].

Pathophysiological consequences of Se deficiency derive from dysfunction of many SePs with important roles in antioxidant defense, cell signaling and transcription, metabolism of thyroid hormones, and in immune and reproductive systems [44,65]. Se and vitamin E play complementary but independent functions as cellular antioxidants, which protect cells from injury by reactive oxygen species generated during normal oxidative cellular metabolism. Se is a component of glutathione peroxidase, a family of selenoenzymes that inactivates peroxides thus preventing membrane damage; vitamin E acts as free radical scavenger, providing protection against peroxidation of polyunsaturated fatty acids [44,45,65]. Failure of antioxidant mechanisms caused by Se/vitamin E deficiency leads to intracellular accumulation of free radicals with subsequent membrane damage and intracellular influx of calcium (Ca^2+^); this is first moved from the cytosol to the mitochondria, causing damage in the latter, and ATP depletion. Eventually, raised Ca^2+^ cytosolic levels result in myofibril hypercontraction and cellular necrosis with secondary dystrophic mineralization [44,66,67]. Cells or tissues that undergo rapid increases in oxidative metabolism, such as striated muscle and red blood cells, are particularly susceptible to the oxidative injury that underlies NM, Heinz-body anemia, and reproductive failure related to Se/vitamin E deficiency in sheep [44,66,67]. In addition to oxidative damage, dysfunction of a series of deiodinases involved in the synthesis of thyroid hormones are also important contributors to growth retardation and infertility problems in Se-depleted lambs and sheep [44,65,68].

The most classical clinical manifestation of Se/vitamin E deficiency in sheep is NM, although many cases are related to Se deficiency, only. The incidence of NM may vary from 1 to 30% depending on the geographical region. It primarily affects young lambs and presents as two major clinical forms, which can develop in- or ex-utero. The congenital form is rare, but may be associated with high mortality; stillbirths or extremely weak neonates occur, and the latter usually die within a few days, often from acute cardiac arrest [45,66]. Delayed or acquired forms are usually encountered in 2–6-week-old lambs that have recently turned out onto the first green pasture, but they can also occur in younger or older animals [44,45]. Mortality rates in outbreaks among lambs from one day to two months may be low or reach 50%. A second peak of incidence, frequently with lower mortality but moderately higher incidence of subclinical or minimal forms, occurs at 4–8 months of age, when weaned lambs are put onto lush pastures or feedlots. In mature sheep, clinical manifestations are sporadic, but subclinical disease may involve 5–30% of a group [66]. The general signs are similar in all cases and include weakness, stiffness, rapid breathing, and deterioration of muscles, usually starting in the hind limbs. Indeed, hindquarters are often swollen, lambs exhibit an unsteady and painful gait, and adopt an antalgic posture [45,66]. Older lambs may suffer respiratory distress, often with secondary pneumonia, which complicates the diagnosis [66,67]. The severity of the signs may range from mild discomfort to recumbency or collapse and, eventually, lambs lose body condition, become prostrate, and usually die [44,45]. Additional stressors such as bad weather, prolonged winter feeding, forced activity, hypocuprosis, and other management procedures, can dramatically exacerbate the incidence and/or severity of the disease [44,45,66].

Se deficiency may also cause unspecific signs in weaned lambs and hoggets with important effects on productivity, which can appear without evidence of clinical or subclinical cases of NM [44,45,65]. Se-responsive ill-thriftiness can range from subclinical wool and body growth deficits to visible emaciation and poor external aspect. In extreme cases, death may ensue. Heinz body anemia has also been seen in Se deficient lambs stressed by exercise [44,45,65]. In ewes, infertility problems and decreased reproductive performance have been attributed to Se inadequacy [44,69].

The gross appearance of muscular lesions in NM depends on the extent of the necrosis and the chronicity of the process, and are often difficult to see in mild cases [45,67]. In severe cases, muscular lesions are easily visible as pale streaks parallel to fiber direction, which become chalky due to mineralization at chronic stages [66,67]. As muscles in lambs are usually pale, the recognition of the mineralized flecking is almost essential if diagnosis is to be made grossly [66]. Cardiac involvement is frequent, particularly in the congenital form, and lesions are readily observed beneath the epicardial and endocardial surfaces [45,70]. Muscular lesions in lambs are bilateral and symmetrical and most commonly involve major muscles of the thigh and shoulder, but others can also be affected. In old sheep, lesions might be restricted to the intermediate head of the triceps or to the tensor fascia latae muscle and, in pregnant ewes, to the abdominal muscles, which may rupture allowing viscera to herniate. In the congenital form, the most severe lesions frequently involve the tongue and neck muscles, and may be detectable in fetuses at least two weeks before parturition [45,66,70]. Histologically, muscular lesions consist of a selective, segmental, and polyphasic myofiber degeneration and necrosis, which predominantly involves type I fibers [66,67]. Myocytes show sarcoplasmic swelling and vacuolation or hyalinization, loss of cross striations, fragmentation, and occasional contraction bands. Additional features include foci of dystrophic mineralization, macrophage infiltration, satellite cell proliferation, and signs of myocyte regeneration [66,67,71].

The diagnosis of Se-responsive ill-thriftiness is difficult because the subclinical/clinical and pathological consequences are frequently unspecific, the pathogenesis is multifactorial, and biochemical confirmation is rather complicated [44,45]. Therefore, in some cases the diagnosis and the effects of Se deficiency can only be established by tissue analysis in combination with a response trial [44,45]. Diagnosis of NM is based on clinical, morphological, and histological alterations, but confirmation requires evidence of Se/vitamin E deficiency in blood or, alternatively, in tissues (i.e., liver, kidney) [44,45,66]. Decreased glutathione peroxidase activity in blood may be a more reliable indicator of low Se status than blood Se, particularly in neonates; however, it occurs slowly [44,72,73]. For a clinical diagnosis, biochemical markers of muscular injury, such as creatine kinase and troponin I, are useful to confirm muscular damage and discriminate between skeletal and cardiac involvement [44,52,74].

Clinical and subclinical cases of NM are routinely treated by subcutaneous injections of Se/vitamin E mixtures. Risk of Se toxicity should be taken into account in severely affected animals that require weekly treatments along several weeks [44,45,75]. Se deprivation can be prevented by supplementation in many ways, and the method of choice depends on the husbandry conditions [44,45]. Subcutaneous injections of Se/vitamin E are widely used prophylactically in ewes during late pregnancy and in newborn or weaning lambs [44,45,69,76]. Periodic oral drenching with Se salts is also effective. Slow-release Se pellets or injections are useful for continuous, long-term supplementation, but application could be difficult in young lambs, and may provide variable and limited protection to older sheep [44,45]. Vitamin E supplementation is particularly important during early periods of pasture growth due to higher contents in polyunsaturated fatty acids, and in diets with expected low vitamin E content. Combined Se/vitamin E supplements can sometimes be conveniently and efficiently offered with the diet [44,45].

### 3.4. Zinc Deficiency

Zn is an essential nutrient and plays an important role in many metalloenzymes that regulate metabolic processes, tissue growth, maturation, and repair [44,51]. Zn also modulates many aspects of the immune and inflammatory responses because of its structural role in the vast majority of transcription factors involved in immune system development [46,77].

Features of Zn deficiency include loss of appetite and growth retardation, reproductive disorders, depression of the immune response, hematologic abnormalities, impairment of central nervous system development, decreased wound healing, alopecia and keratinization defects in epidermis, hair, wool, and horny appendages [46,51,78].

Loss of appetite is an early sign of Zn deprivation and there are changes in the patterns of food intake from ‘meal-eating’ to ‘nibbling’ in ruminants [44]. Zn deprivation increases the expression of the gene that encodes for the appetite-regulating hormone cholecystokinin, which induces satiety [79]. There is also increased expression of leptin, a cytokine hormone whose secretion from adipocytes also promotes a satiety signal [80]. This reduction in appetite is selective, with carbohydrates being avoided and proteins and fats preferred [44].

Zn deprivation in pregnant ewes does not cause congenital malformations, although the number of born lambs and their weights may be reduced due to restricted fetal growth [44]. Diets very low in Zn during pregnancy reduce survival of the newborn lamb, and pregnancy toxemia may occur as a secondary consequence of anorexia in the ewe [44]. Testicular hypoplasia occurs experimentally in Zn-deprived male lambs [81]. In that experiment, spermatogenesis almost entirely ceased within 20 weeks on a diet containing very low amounts of Zn, but recovered completely during a repletion period.

Zn has many roles in immunity and disease resistance as well. In a study, there was a reduction in the percentage of lymphocytes in the peripheral blood of lambs fed with a Zn-deficient diet [82]. Animals with hereditary Zn deficiency have a hypoplastic thymus and, consequently, secondary infections are common because of associated immune system dysfunction involving both humoral and cell-mediated immunity [44,45,83].

Thickening, hardening, and fissuring of the skin due to hyperkeratosis is a late sign of Zn deprivation in all species [51]. In lambs, frequently affected sites include periocular areas, nose, coronary band, scrotum, and pressure points [51]. Lesions can also affect stratified epithelia lining the tongue and esophagus, and are similar to those induced by vitamin A deficiency [44]. Hyperkeratosis also affects the forestomachs [51]. The rate of healing of skin wounds is retarded in Zn-deprived animals, and wounds caused by ectoparasites or other skin infections will probably exacerbate the effects of hyperkeratosis [78]. In horned lambs, the normal ring structure disappears from new horn growth, and the horns are ultimately shed, leaving soft spongy outgrowths that bleed continuously [44,51]. Abnormal hoof growth may lead to soreness and the adoption of a kyphotic stance [51]. Sheep also have thin, easily epilated, redbrown colored wool and loss of fleece. Wool eating and drooling are prominent clinical signs [51,78]. The characteristic histologic lesion is severe and diffuse hyperkeratosis. In sheep with naturally occurring Zn deficiency, the hyperkeratosis is predominantly of the orthokeratotic rather than the parakeratotic type [51]. In addition, skin from Zn-deficient sheep contains apoptotic bodies in follicle bulbs and the wool fibers are improperly keratinized as indicated by retained cell nuclei and fiber distortion in the distal parts of some follicles [78].

Severe Zn deficiency can readily be diagnosed from the combined evidence of clinical and pathological disorders and the biochemical defects, but the diagnosis of early stages or milder forms presents difficulties [44]. The diagnosis of Zn deprivation in sheep can be done by using a lower limit of normality of 0.6 mg (10 mmol) Zn/l in serum [44]. Serum alkaline phosphatase activities decrease in the Zn-deprived lamb [44]. Bone, hair, wool, and feathers are particularly rich in Zn, and concentrations in the appendages of clinically affected animals are reduced. However, individual variability is high and values are affected by age, breed, sampling site, and season [44,78].

In housed livestock, Zn deprivation can be prevented readily and cheaply by adding Zn to mineral supplements or total mixed rations [44]. Other methods are available in grazing sheep. For instance, treatment of soil with Zn containing fertilizers is feasible, but amounts may vary with the environment. In extensive systems, with broad pastures, fertilizer applications may be uneconomic, and the provision of salt licks containing 1–2% of Zn should provide sufficient cover [44]. Supplements should be used regularly because Zn is not stored well in the organism. An intra-ruminal Zn pellet can release sufficient Zn to overcome seasonal deficiencies. Supplementation with “organic” as opposed to inorganic sources of Zn offers no consistent nutritional or health advantage for sheep [45].

### 3.5. Iodine Deficiency

Iodine (I) has only one known, though vital, function since it is an essential constituent of thyroid hormones. Thyroid hormones have a thermoregulatory role, increasing cellular respiration and energy production, as well as widespread effects on intermediary metabolism, growth, muscle function, immune defense, and circulation [45,84,85]. These are particularly important in facilitating the change from fetal to a free-living stage [44]. Seasonality of reproduction in ewes is related to changes in thyroid activity [86].

Iodine deficiency disorder (IDD) may arise from simple dietary lack or due to low content in the soil [44]. IDD is also induced by exposure to goitrogens contained in brassicas, white clover, or legume crops. These act by disrupting I metabolism, either by impairing its uptake by the thyroid gland, or by promoting iodination of tyrosine residues [45]. However, widespread addition of iodized salt to animal diets prevents most of the outbreaks nowadays, and fewer animals tend to be affected if they occur [85].

Goiter (enlarged thyroid glands) is a clinical manifestation of IDD [44,87]. Goiter is manifested predominantly in the newborn animal, which is usually delivered by a clinically normal dam [44]. In sheep, insufficient I intake by pregnant ewes and subsequent fetal hypothyroidism results in late term abortions, increased perinatal mortality, and birth of weaker lambs with visibly enlarged thyroids and delayed skin and wool follicle development [44,85,87]. Delayed bone growth and maturation may also occur if the ovine fetus is exposed to I deficiency up to the last trimester of gestation [87]. Gestation is often prolonged, and most severe cases of thyroid enlargement may cause dystocia, with a tendency to retain the fetal placenta [85]. The degree of thyroid enlargement increases with the level and duration of I deprivation. Size tends to be indicative of compensatory attempts for insufficient thyroid hormone production [44]. Asphyxiation may also result from pressure by the enlarged gland on the trachea and adjacent structures [85]. Retarded CNS development has been observed in severely depleted fetuses, and behavioral abnormalities may thus ensue after birth [45].

Flocks lambing in spring, particularly in years of increased winter–spring rainfall, are usually at higher risk of IDD [45,87]. Environmental conditions in that particular time of the year lead to increased pasture growth and leaching of the mineral from topsoil, both of which may reduce the intake of I by grazing animals.

Less severe goiter without any apparent ill-effects on health and production may also occur with grazing on various brassicas [45,87]. Fetal development may be arrested at any stage by thyroid dysfunction, leading to early death and resorption, abortion, or subnormal birth weights [44,88]. The need for thyroid hormones to produce lung surfactants may be an important determinant of neonatal survival [84].

Older animals are rarely clinically affected, although infertility, deterioration in semen quality and loss of libido in the male, poor wool growth, depressed milk yield, and reduced weight gain have been recorded [44,45]. Hill (1991) [89] reported hypothyroidism induced by the prolonged feeding of food rich in goitrogen substances is accompanied by loss of appetite leading to impaired growth and/or depressed milk yields. Milder deficiency is reflected in minor changes, such as scanty wool and hairiness of the fleece in sheep [87]. Thyroid insufficiency in the young lamb permanently impairs the quality of the adult fleece, since the normal development of the wool-producing secondary follicles is highly dependent on thyroid activity [44].

Gross findings in the affected lambs include markedly enlarged, homogeneously firm, dark maroon-colored thyroid glands. The tongue is usually swollen and there is edematous swelling of the fauces and larynx, which are possible contributory factors to death [85,87]. Both thyroid lobes are uniformly enlarged. There is frequent myxedema and alopecia in newborn lambs [44,85,87]. The major histopathological findings are seen in thyroid structure of the I-deficient, newborn lambs and consist of a marked hypertrophy and hyperplasia of the cuboidal epithelium of the thyroid follicles, with little or no colloid production, and follicular collapse. These changes define the so-called hyperplastic goiter [44,85]. Immature, poorly-differentiated wool follicles and sebaceous glands may be detected in lambs with areas of alopecia. The epiphyseal ossification centers of the long bones can be either very small or absent, reflecting an age-inappropriate delay in development [85,87].

Later in time, a form known as colloid goiter occurs. It represents the involution phase of hyperplastic goiter and is usually seen in young adult and adult animals. The markedly hyperplastic follicular cells continue to produce colloid, but endocytosis is decreased due to diminished pituitary TSH levels in response to the return of thyroxine and triiodothyronine levels to normal. Both thyroid lobes are diffusely enlarged, but are more translucent and lighter in color than with hyperplastic goiter due to reduced vascularity and development of very distended macrofollicles [85].

Diagnosis of I deficiency is based on gross and histological findings combined with biochemical indices of I status. Severe goiter can be easily diagnosed from a visibly or palpably enlarged thyroid, but milder forms require determination of thyroid weight [44]. Thyroid gland to body weight ratios higher than 0.9 g/kg are indicative of goiter. Total thyroid weight higher than 2.8 g has been used as a diagnostic threshold for congenital goiter [44,45]. In the bloodstream, serum T4 decreases, thus causing a reduction in the serum T4 to T3 ratio [44]. Values for normal serum T4 of >47 and >90 nmol/L have been used as thresholds to diagnose I sufficiency in ewes and lambs, respectively, while plasma T4 concentrations of <20 nmol/L, and pasture I contents <0.09 mg/kg have been associated with high incidence of congenital goiter [45]. Urinary and milk I concentrations are also good markers of I intake. Milk is an important route of I secretion, and concentrations below 0.55 µmol/L are indicative of dietary deficiency [44,45].

IDD caused by simple dietary deprivation can be prevented and cured by I supplementation (i.e., using oral boluses and drenches), most often in the form of iodide. Intramuscular injection of iodized poppy-seed oil is perhaps the most effective treatment [45]. Iodine injections can reduce goiter incidence and improve survival to weaning, increase wool yields in the year after treatment, and control goiter in the next lambing season [44]. IDD caused by goitrogens require other measures. In hand-fed animals, supplementation is accomplished by the use of iodized salt licks, kelp (seaweed)-based compounds, and/or by incorporating I into mineral mixtures or concentrates [44,45]. Animals in I-deficient areas that have grazed abundant pasture based on cereals and root crops may also require supplementation, particularly in late pregnancy [45,87].

## 4. Conclusions

This review emphasizes that a correct nutritional management is a key factor to prevent wasting in sheep flocks. Feed has to be offered not only in enough amount, but also with a qualitative composition that ensures an appropriate intake of all the necessary minerals, vitamins, and other micronutrients. In addition to emaciation and loss of performance, some mineral deficiencies produce lesions that may facilitate a tentative diagnosis. Such diagnosis must be confirmed by demonstrating low levels of the suspected imbalanced nutrient in organs or body fluids. Restituting these depleted levels is the therapeutic approach to take in most cases, either by selecting feeds with optimal amounts of such nutrient/s or by using supplements and mineral correctors.
